# Immune receptor mimicking hormone receptors: a new guarding strategy

**DOI:** 10.1007/s44154-023-00095-0

**Published:** 2023-05-26

**Authors:** Xueru Liu, Josh Li, Tony ShengZhe Peng, Xin Li

**Affiliations:** 1 Michael Smith Laboratories, University of British Columbia, Vancouver, BC V6T 1Z4 Canada; 2 Department of Botany, University of British Columbia, Vancouver, BC V6T 1Z4 Canada

**Keywords:** Plant immunity, Effector, NLR, Phytohormone receptors, LRR, TSWV

## Abstract

Plant intracellular nucleotide-binding domain leucine-rich repeat (NLR) receptors play crucial roles in immune responses against pathogens. How diverse NLRs recognize different pathogen effectors remains a significant question. A recent study published in *Nature* uncovered how pepper NLR Tsw detects phytohormone receptors’ interference caused by *tomato spotted wilt virus* (TSWV) effector, triggering a robust immune response, showcasing a new manner of NLR guarding.

## Main text

The plant immune system relies on immune receptors to recognize pathogen components and trigger defense responses. The superfamily of nucleotide-binding domain leucine-rich repeat (NLR) proteins represents a major group of immune receptors in higher plants that evolved to detect the presence of pathogen effectors delivered into host cells. Although a few studies reported direct recognition of effectors by NLRs, indirect recognition such as guard, decoy, or integrated decoy models, seems to occur more prevalently (van Wersch et al. 2020). These indirect recognition strategies enable broad-spectrum pathogen recognition and help plants keep up with the rapid evolution of pathogens. Plant NLRs typically have variable N-termini, a central nucleotide-binding domain and a C-terminal leucine-rich repeat (LRR) region. The LRR often serves important roles in effector recognition and self-inhibition (Jones et al. 2016; Maruta et al. 2022). Tsw, an NLR from pepper *Capsicum chinense*, was known to confer resistance against *tomato spotted wilt virus* (TSWV). This Tsw-mediated immunity is activated by the effector NSs, a gene-silencing suppressor protein encoded by TSWV (de Ronde et al. 2013). What is intriguing is that the LRR domain of Tsw is over twice as large as a canonical LRR from NLRs. Chen and colleagues (Chen et al. 2023) thus raised the question why pepper Tsw has such huge LRR, and what its function might be.

**Fig. 1:**
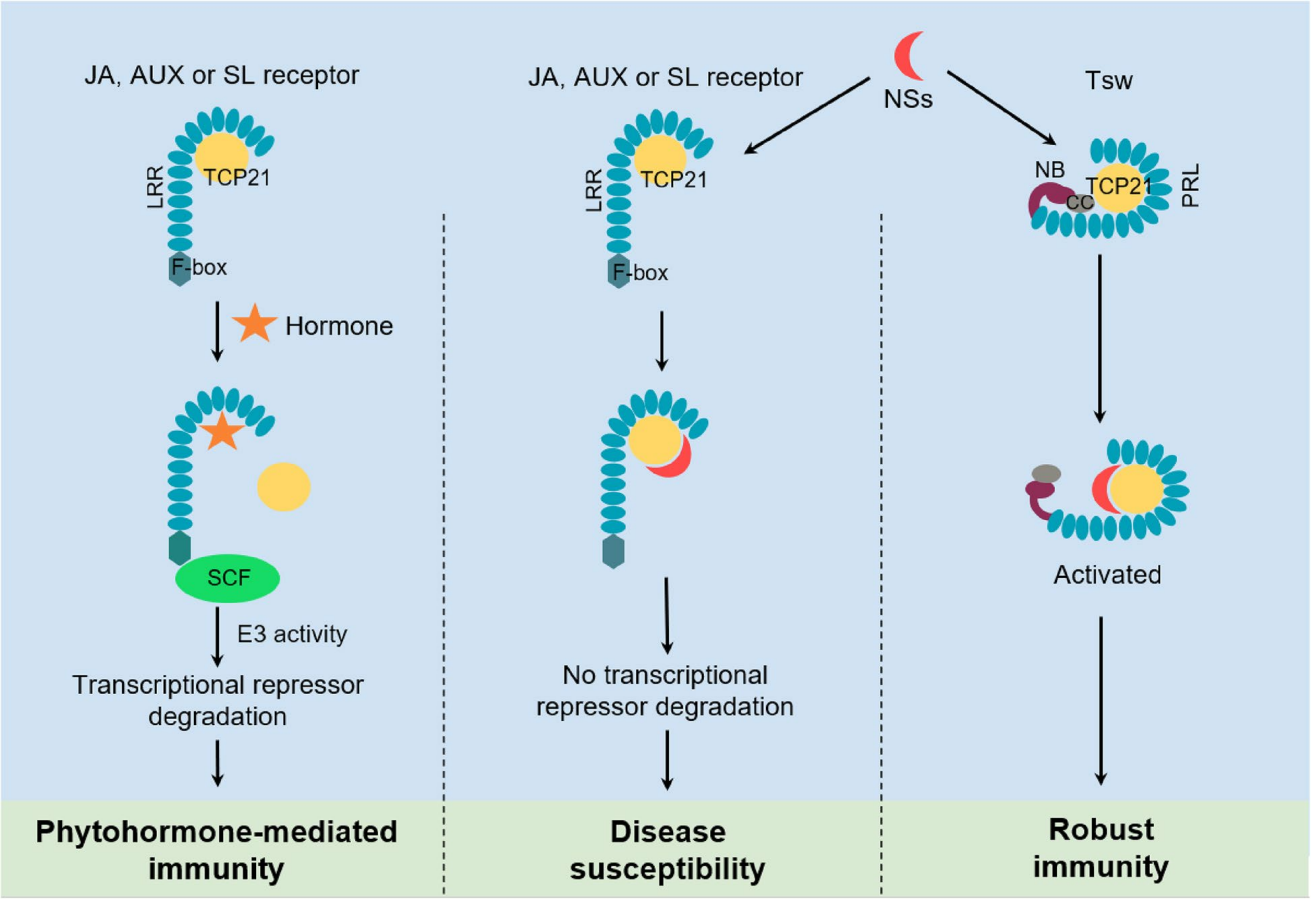
NLR Tsw detects interference of phytohormones by TSWV effector NSs and triggers immunity. The F-box-type JA, AUX, or SL phytohormone receptors trigger immunity upon binding of the active hormone molecule to the LRR domain. Subsequently, the formation of the Skp1/Cullin/F-box (SCF)-containing E3 ubiquitin ligase complexes target the downstream transcriptional repressors for ubiquitination and degradation, activating immune responses mediated by these hormones. TSWV encodes effector NSs to target the phytohormone receptor LRR domain through TCP21, preventing the transcriptional repressor degradation and promoting susceptibility to TSWV. However, plants have evolved NLR Tsw with a similar LRR domain as the phytohormone receptors—phytohormone receptor-like (PRL) domain, to detect NSs also through TCP21, which in turn activates a robust immune response

To gain a better understanding of the LRR domain of Tsw, Chen and colleagues first conducted 3D structural modeling and homology comparison to identify proteins with a similar LRR structure. Interestingly, they found that the LRRs of three phytohormone receptors: jasmonic acid (JA) receptor Coronatine-insensitive 1 (COI1), auxin (AUX) receptor Transport inhibitor response 1 (TIR1), and strigolactone (SL) receptor partner More Axillary Growth2 (MAX2), exhibit great structural similarities to part of the Tsw LRR domain, which was then named as phytohormone receptor-like (PRL) domain. The phytohormones JA, AUX, and SL have central roles in regulating various physiological processes, not only in plant growth and abiotic stress, but also in regulating immune responses against different pathogens (Berens et al. 2017). They are perceived by the respective receptors to transduce the signal to downstream components. Coincidentally, JA, AUX, and SL receptors are all LRR-containing F-box-type protein receptors, functioning as part of the Skp1/Cullin/F-box (SCF) E3 ubiquitin ligase complex. The recognition of phytohormones by their receptors can promote the ubiquitination and degradation of the downstream transcriptional repressor proteins. This allows de-repression of transcription factors to activate transcription (Takeuchi et al. 2021) (Fig. 1). The precise regulation of these hormone signaling pathways is not only essential for normal plant growth and development, but also allows plants to mount rapid and effective responses against pathogens. However, pathogens have evolved diverse strategies to interfere with these signaling components to promote virulence (Han and Kahmann 2019). As pepper NLR Tsw recognizes effector NSs to activate immunity and Tsw has a similar LRR structure as JA, AUX, and SL receptors, Chen and colleagues hypothesized that NSs may target phytohormone receptors to promote its virulence. During an evolutionary arms race, pepper evolved NLR Tsw with a highly similar PRL domain as the phytohormone receptors. Tsw mimics these hormone receptors to be targeted by NSs, thus activating a robust defense response to restrict pathogen infection.

To test whether NSs interferes with JA, AUX, or SL signaling, Chen and colleagues first generated an Arabidopsis transgenic line overexpressing NSs. JA, AUX, and SL levels as well as their responsive genes’ expression were significantly downregulated by ectopic NSs expression. Further biochemical analysis revealed that NSs associates with JA, AUX, and SL receptors COI1, TIR1, and MAX2, but not directly. From a yeast-2-hybrid screen using NSs as the bait, several Teosinte branched1/cycloidea/proliferating cell factor (TCP) members were identified. Among them, TCP21 not only showed interaction with NSs, but also with COI1, TIR1, and MAX2, suggesting that NSs targets these receptors through TCP21 (Fig. 1). In agreement with this, overexpression of TCP21 had similar effects as NSs, negatively regulating JA, AUX, and SL responsive genes’ expression. Further analysis showed that TCP21 can dampen the interactions between F-box-type receptors COI1, TIR1, and MAX2 and their transcriptional repressors Jasmonate Zim Domain (JAZ), Auxin/Indole-3-acetic Acid (Aux/IAA), and Suppressor of MAX2-like (SMXL). This results in a delay in the degradation of transcriptional repressors, ultimately preventing the activation of downstream transcriptional reprogramming. Moreover, the presence of NSs strengthened the interactions between TCP21 and COI1, TIR1 and MAX2, which further attenuated the association of COI1, TIR1, and MAX2 with JAZ, IAA, and SMXL, and delayed the degradation of JAZ, IAA, and SMXL, leading to significant downregulation of JA, AUX, and SL signaling. Based on the structural similarities between COI1, TIR1, and MAX2 with Tsw, the authors tested the interaction between TCP21 and Tsw PRL domain. Interestingly, TCP21 interacted with Tsw PRL and this association was enhanced by NSs. However, relative to COI1, TIR1, and MAX2, TCP21 has greater affinity with Tsw in the absence of NSs and even better affinity with Tsw in the presence of NSs. This indicates that NSs interferes with phytohormone receptors via TCP21, and pepper uses NLR Tsw to detect NSs through TCP21, subsequently activating a robust defense response to limit virus growth (Fig. 1).

In this study, by using the amino acid sequence of Tsw for a homology search, there was no indication of the function of the unusually large LRR domain of Tsw. However, the structural comparison search identified highly similar phytohormone receptors, which provided a solid foundation for generating the hypothesis of this study. In recent years, with the rapid development of the Alphafold protein structure database, millions of high-quality protein structures are available online (Jumper et al. 2021). Researchers can now expand their search beyond the traditional sequence BLAST to structure ‘BLAST’, which offers higher sensitivity to identify functional homologues of proteins of interest, providing deeper insights into protein function.

In addition, this study offers a new model for NLR activation during plant defense response. Previous studies suggested some indirect recognition models. In the guard model example, Arabidopsis NLRs RPM1 (resistance to *Pseudomonas syringae pv. maculicola* 1) and RPS2 (resistance to *P. syringae* 2) guard the effectors targeting guardee RIN4 (RPM1 interacting protein 4) (Mackey et al. 2002, 2003). In the integrated-decoy case, where the bacterial effectors target plant WRKY transcription factors to promote virulence, NLR RRS1 (resistance to *Ralstonia solanacearum* 1) carries an integrated WRKY domain to capture the effectors, activating defense (Le Roux et al. 2015; Sarris et al. 2015). This new study shows that plant NLRs can also carry a similar PRL domain mimicking the effector-targeted phytohormone receptors to detect effectors. This provides a new avenue to search for unknown effector-targeted plant signaling components. It will be interesting to investigate whether there are any other NLRs using the same strategy to detect the interference of effectors and plant hormones. Another notable finding from this study is that the effector NSs targets a common repressor protein TCP21 to interact with all three phytohormone receptors, enlarging the range of the host proteins to be targeted. Tsw also detects NSs through TCP21. Other proteins that can interact with TCP21 will be of interest to enrich our understanding of the molecular mechanisms of TCP21 in immune regulation. It will also be interesting to study how the structure of Tsw PRL domain determines its higher affinity with TCP21 and effectors than the phytohormone receptors.

In summary, Chen and colleagues identified how NLR Tsw detects the interference of phytohormone receptors by TMSV effector NSs through the structurally similar PRL domain. This discovery improves our understanding of how NLRs are activated by pathogens. The novel PRL domain in NLR could potentially be employed to engineer NLRs to improve crop disease protection.

## Data Availability

Not applicable.

## Abbreviations

LRR: Leucine-rich repeat
NLR: Nucleotide-binding domain leucine-rich repeat receptor
TSWV: *tomato spotted wilt virus*
JA: Jasmonic acid
COI1: Coronatine-insensitive 1
AUX: Auxin
TIR1: Transport inhibitor response 1
SL: Strigolactone
MAX2: More axillary growth 2
PRL: Phytohormone receptor-like
SCF: Skp1/Cullin/F-box
TCP: Teosinte branched1/cycloidea/proliferating cell factor
JAZ: Jasmonate Zim Domain
Aux/IAA: Auxin/Indole-3-acetic Acid
SMXL: Suppressor of MAX2-like
RPM1: Resistance to *Pseudomonas syringae pv. maculicola* 1
RPS2: Resistance to *P. syringae* 2
RIN4: RPM1 interacting protein 4
RRS1: Resistance to *Ralstonia solanacearum* 1
